# Meta-Representational Skills in Bullying Roles: The Influence of Definitional Competence and Empathy

**DOI:** 10.3389/fpsyg.2020.592959

**Published:** 2020-10-22

**Authors:** Carmen Belacchi, Beatrice Benelli

**Affiliations:** ^1^Department of Communication Sciences, Humanities and International Studies, University of Urbino Carlo Bo, Urbino, Italy; ^2^Department of Developmental Psychology and Socialization, University of Padova, Padua, Italy

**Keywords:** meta-representational ability, metalinguistic skills, empathy, bullying roles, primary school children

## Abstract

This study investigated the influence of meta-representational aspects on bullying. Meta-representation was operationalized in terms of the metalinguistic skill to produce conventional definitions, reflecting culturally shared representations and of the meta-level capacity to represent others’ mental states underlying empathic disposition. One hundred and seventeen children, aged between 8;5 and 10;11 years, completed a definitional task and self-report questionnaires on bullying roles and empathic disposition. Descriptive, correlational, and regression analyses were performed. Results confirmed that hostile roles are negatively related to definitional competence and to empathic disposition. Lack of definitional competence was the main predictor (accounting for about 16% of variance), followed by empathy (explaining a further 6% of variance) of Primary School children’s disposition to assume aggressive behaviors. These findings suggest that a lack of general meta-representational abilities may hinder the development of abstract and other-centered perspective taking, and compromise (compromising) social adjustment. This implies the need to work, particularly in school, on enhancing meta-representational and metalinguistic skills, such as the ability to recognize mental states and verbally make explicit cultural-semantic word meaning representations.

## Introduction

The present contribution aims to understand the relationship between the metalinguistic capacity to provide conventional word definitions, empathic disposition, and children’s behavior in bullying episodes. Although language and empathy are different in nature, it is reasonable to assume that they share high-level cognitive and meta-representational components: producing good definitions demands the metalinguistic capacity to follow conventional rules for making word meaning explicit, the selection of relevant semantic content components, and the use of appropriate linguistic forms; empathy requires the ability to analyze other people’s mental states and compare them with one’s own, without confusing the perspectives of self and others.

A lack of reflective, meta-representational capacity in both these areas may be associated with aggressive conduct, in terms of impulsive, automatic reactions in social contexts, especially threatening ones. In the bullying literature, the connection between empathic disposition and aggressive behaviors has already been extensively studied, while less attention has been devoted to the role of language in regulating social conduct and interpersonal episodes.

### Bullying and Other Hostile Social Roles

Bullying has conventionally been defined as: “repetitive negative actions intended to harm or cause significant distress, inflicted by a more powerful person against a less powerful one” (Jolliffe and Farrington, 2006, p 540). It is basically a group phenomenon: since Salmivalli et al. (1996), a significant body of research has demonstrated the existence of different roles among children and adolescents involved in bullying episodes: the Bully, who actively performs negative social actions; the Assistant, who helps the Bully; the Reinforcer, who without directly intervening supports the aggressive actors; the Defender, who actively helps the victim; the Outsider, who does not take part in the aggressive episodes in any way, and the Victim. More recently, Belacchi (2008) identified two other roles: the Mediator, who actively attempts to reconcile the Bully and Victim, indirectly supporting the latter, and the Consoler who, without directly intervening in the situation, tries to mitigate the effects of the bullying by comforting the Victim; both of these roles are related to the Defender role and more analytically operationalize the spontaneous pro-social tendency to intervene in interpersonal conflicts among peers in a group setting. In this model, the Outsider role is positively associated with hostile roles and negatively with pro-social ones, confirming the essentially aggressive attitude of Outsiders.

### Nature and Forms of Meta-Representation

Generally speaking, meta-representation can be defined as the capacity to conceive the existence of different mental level at which a given kind of information can be cognitively coded and analyzed. This implies that each kind of competence or performance (cognitive, linguistic, and social) takes different forms, from the natural, spontaneous and most primitive ones to the most mature, reflected and conscious expressions (Karmiloff-Smith, 1992). In other words, in this process, a given mental faculty shifts from being a “tool for thinking” to being an “object of thought.” Metacognition is the awareness of one’s own cognitive skills, processes, strategies, and limits (particularly of memory); metalinguistic awareness is a complex construct that can be operationalized according to the different aspects and functions of the language itself: as capacity to identify and deal with the linguistic sounds (meta-phonology); as understanding of the formal rules of language (meta-syntax); of the appropriate linguistic codes to be used in the different social contexts (meta-pragmatics); and meta-semantics, that is, the awareness of the arbitrary nature of the linguistic system (For these distinction see: Gombert, 1990). What all these dimensions have in common is that the focus is no longer on the content of what is said (as in the colloquial use of language) but rather on the form of the message, that is, the way in which it is said. The most representative example of metalinguistic competence are definitions: being a de-contextualized task, definitions cannot rely on environmental and expressive cues to convey the intended meaning, thus forcing the speaker to accede to the most general and intrinsic aspects of the conventional, culturally shared word meanings (e.g., Benelli et al., 2006).

As regards the meta-representational nature of empathy, this issue has been scarcely empirically investigated but often implicitly assumed, in order to properly identify its different evolutionary forms. In fact, in the capacity to understand mental states, and in the awareness of its causal effects on the self some kind of meta-knowledge can be seen (Walter, 2012), which distinguishes the more mature empathic reactions from emotional contagion based on automatically triggered reflexes (Custance and Mayer, 2012). According to Blair (2005) – who distinguishes between cognitive, affective, and motor empathy – cognitive empathy is used where the individual represents the internal mental state of another individual; in this sense, cognitive empathy is equated to Theory of Mind (ToM), that is, comprehension that people have beliefs, intentions, desires, emotions, etc., and that their actions are driven by such mental states. Emotional empathy is formed of both a response to the emotional display of another person (e.g., facial and vocal expressions) and a response to other emotional stimuli, (e.g., a phrase such as “Adam just lost his house”). Cognitive empathy requires that information is held in mind and manipulated, on the basis of visual, auditory, or situational cues, representing another person’s cognitive and emotional state. “This process of representation can take place at an explicit level, but it can also arise at an implicit, higher-order level as meta-representation” (Reniers et al., 2011, p. 84): these authors stress that generating ideas about another person’s cognitive or emotional state involves a dynamic working model that requires shifting attention back and forth in order to compare, contrast, or align one’s own cognitive and emotional state with that of the other person. The complex empathy dispositions are underpinned by specific neural systems (e.g., Decety and Jackson, 2004).

As regards the role of empathic disposition in bullying, many authors have confirmed a clear negative link between hostile roles and empathy in school age children (Gini et al., 2007, 2008; Belacchi, 2008). In preschoolers, Belacchi and Farina (2012) found both affective and cognitive empathic disposition to be negatively correlated with the hostile roles.

### Language and Social Development

Language is a powerful means of representation that organizes world knowledge into permanent and systematic mental structures, and enables the sharing of this knowledge among all the members of a given culture. Language also facilitates continual analytical reflection on experience and allows different hypothetical scenarios to be represented. From a communication point of view, language permits the negotiation of meanings, plans, and solutions, as well as the explanation of personal mental states, thus facilitating peer acceptance and social inclusion (Hemphill and Siperstein, 1990).

The role of language in the typical social development has been analyzed by several authors, all of them sharing the assumption that children build their knowledge of the environment, particularly of the social one, by sharing their everyday life with other people, adults, and peers. According to Carpendale and Lewis (2004, p. 79): “the development of children’s social understanding occurs within triadic interaction involving the child’s experience of the world as well as communicative interaction with others about their experience and beliefs (Chapman 1991, 1999).” This process is considered one of the most powerful means to form the child’s Theory of Mind. Expressions such as “I think,” “I know,” “I want,” followed by consequential observable behaviors, contribute to the understanding that people may have different and even conflicting points of view and needs, which therefore must be confronted, mediated, revised, etc. Children whose mothers use mentalistic terms and, therefore, presumably talk to them about the psychological world (mind-mindedness) are more advanced in understanding beliefs than other children (e.g., Meins and Fernyhough, 1999). Particularly the emotional terms (sad, ashamed, angry, envious, etc.) foster comprehension of the complex nature of emotions (Ornaghi and Grazzani, 2013), the regulation of which has a strong impact on interpersonal conducts.

As regards the disadjusted social development, the role of linguistic competence has been extensively explored in the literature on criminal activities: in general, registered criminals, young offenders, deviant adolescents, and drug users have all been found to display poor linguistic ability. For example, Brownlie et al. (2004) found that at 19 years there was a direct effect of language impairment at five on adolescent delinquency, even after controlling for verbal IQ. Snow and Powell (2008) reported that over half of a sample of young male offenders displayed significant deficits on figurative/abstract language, sentence repetition, and narrative language skills; these difficulties could not be explained by low non-verbal IQ. In this kind of literature, the role played specifically by language skills cannot be thoroughly identified, given that, according to its approach, linguistic difficulties co-vary with other developmental problems or adverse life conditions, such as low SES, neglect, or abuse, learning disabilities, and poor academic achievement etc., which clearly contribute to negative outcomes.

Currently, in the bullying literature, the role of linguistic skills has been studied in terms of impact of poor communicative skills on victimization, and a rather stable link between them has been revealed: linguistic deficits, such as Specific Language Impairments or poor verbal-communicative abilities associated with shyness, increase the risk of being victimized (e.g., Conti-Ramsden and Botting, 2004). A very recent study by Clifford et al. (2019) examined the language production of preschool children with different forms of social withdrawal (reticent, solitary-passive, and solitary-active behavior), revealing that reticent, solitary-passive, and solitary-active children produce less language compared to their non-withdrawn peers.

Instead, the issue of the linguistic abilities of bullies, and their association with aggressive conducts, is less studied and more controversial: for example, according to Bonica et al. (2003), strong verbal skills may induce relationally aggressive strategies (e.g., announcing refusal to play with a mate) between 3 and 5 years of age; but, the study by Clifford et al. (2019) analyzing children engaged in subtypes of aggression (relational, physical, and comorbid) showed that physically aggressive children produce less language compared to non-aggressive children.

This issue needs further consideration: the role of language in socially aggressive conducts has been analyzed mostly at the preschool age level; actually, it should be analyzed in older groups of children, that is, when the typical characteristics of the bullying phenomenon, according to Olweus (1993; repeated, intentional, and power differential) and the identification of the other roles involved (Salmivalli et al., 1996) are clearer and better developmentally established (Jenkins et al., 2017).

Generally speaking, given that bullies do not necessarily display poor academic achievement (Woods and Wolke, 2004) and often possess sophisticated social skills (Sutton et al., 1999), up to now there has been no reason in principle to suspect that they have particular difficulties in the linguistic domain. However, given bullies’ tendency to act impulsively and their poor ability to mentally devise alternative solutions to problems (Crick and Dodge, 1994), it is plausible to hypothesize that their reflective use of language may be lacking to some degree. Moreover, in these kind of studies, language has been considered mainly as a communication tool necessary to regulate on-going social interactions in effective, positive ways. Less interest has been devoted to its function of allowing reflection, cognitive analysis, and planning, in other words, to its meta-cognitive nature. We believe that analyzing how linguistic-metalinguistic skills are specifically involved in aggressive conducts could cast more light on the determinants of antisocial attitudes and behaviors.

### Definitions as Metalinguistic Competence

A traditional method of studying metalinguistic skills is word definitions. Lexicographic word definitions, the prototypical form of which is the Aristotelian “An X is a Y that Z” (e.g., “a dog is an animal that barks”) presuppose cognitive and linguistic abilities in order to analytically express the meanings encoded in the mental lexicon. The most primitive answers tend to stress concrete experiential contents (“dogs bark”), followed by answers simply introducing the categorical dimension (“dogs are animals”) and, later, by more or less effective Aristotelian structures. It is affected by schooling (Snow et al., 1989; Gini et al., 2005) and is related to other linguistic skills such as narrative abilities in preschool years (Chang, 2006).

Most importantly, definitions reflect metalinguistic competence: to define implies knowing not only “what to say” but also “how to say it” (Wehren et al., 1981; Watson, 1985, 1995; McGhee-Bidlack, 1991). Benelli et al. (2006) in a sample of 360 participants, from preschool age to adults with low vs. high educational levels, found a progressive improvement in their definitional performance and its association with the performance on a metalinguistic task. This task asked questions on different aspects of language, from basic issues such as the origin of words to specific questions about the nature of definitions and of literacy. Thus, producing good definitions draws on meta-representational competence, in that they require awareness both of the conventional value of definitions and shared meanings and of interlocutors’ informational needs.

Since Litowitz (1977), the age-related definitional levels were based on diverse criteria, emphasizing content components, the absence/presence of superordinate terms, and the combination of content and formal aspects (Johnson and Anglin, 1995). A new approach (Belacchi and Benelli, 2007, 2017), far from neglecting the role of conceptual contents, places greater emphasis on the syntactic structure of the linguistic responses, starting from the theoretical assumption that only correct and complete forms are apt to account for subjective semantic representations and to share them interpersonally. The rationale of the increasing formal correctness of the response levels is shown in Table 1.

**Table 1:** Definitional levels and corresponding prototypical answers.

Levels	Kinds of answer	Score
1. Non-definitional	No answer or non-verbal answers	0
II. Pre-definitional	One-word answers, mostly associations (e.g., *dog - > tail*)	1
III. Quasi-definitional	Initial formulation of sentences, yielding inappropriate, non-autonomous forms (e.g., *dog - > with a tail; when it barks*)	2
IV. Narrative/descriptive definitional	Formally correct and autonomous sentences, with narrative/descriptive content (e.g., *Dogs bark; Dogs are furry*)	3
V. Categorical definitional	Formally correct and autonomous sentences in categorical/synonymic form, with insufficient content (e.g., *The dog is an animal*)	4
VI. Aristotelian, metalinguistic definitional	Form and content correctness and semantic equivalence (e.g., *A dog is an animal that barks*.)	5

### The Present Study

The first aim of the present study is to analyze the relationships between the disposition to adopt different hostile roles and the ability to define word. The second aim is to cast more light on the underlying meta-representational aspects of empathy, by relating it with the capacity to produce definitions of metalinguistic nature, more than analyze the developmental trends of empathy as such.

The first hypothesis predicted a positive relationship between definitional scores and empathy, consistently with the assumption of a common meta-level of both definitional skills and empathic disposition.

The second hypothesis predicted an increase in scores in the definitional task, as age increases, on the basis of the traditional literature and of previous studies using the present scale.

According to the third hypothesis, a negative relationship between the ability to define words and the tendency to play hostile roles was expected, on the basis of a presumed lack of meta-representational skills in hostile children.

According to the fourth hypothesis about the relationship between empathy and roles, a negative association with hostile roles and a positive association with the pro-social ones was expect, in accordance with the literature and previous studies.

## Materials and Methods

### Participants

The sample comprised 117 Italian children (44% females; age range: 8;5–10;11 years): 36 in the third grade of primary school (*M*_age_ = 8;9, *SD* = 0.30), 42 in the fourth grade (*M*_age_ = 9;9, *SD* = 0.39), and 39 in the fifth grade (*M*_age_ = 10;8, *SD* = 0.40). Pupils attending lower school levels were not considered since the self-report questionnaire requires well-established skills in reading and writing, which cannot be taken for granted in younger children. None of the participants had been diagnosed with any kind of impairment. Participants attended primary schools in a medium-sized city of Central Italy and were from middle-class SES backgrounds, defined in terms of parental levels of education and parental occupations.

The study was conducted in accordance with the ethical standards laid down in the 1964 Declaration of Helsinki and fulfilled the ethical standard procedures recommended by the Italian Association of Psychology. Written informed parental consent, as well as oral informed child assent, was obtained and collected prior to participation, according to the ethical norms in our university.

An *a priori* power analysis has been used to estimate the sample size (using GPower 3.1; Faul et al., 2007). With an alpha = 0.01 and power = 0.95, the projected sample size needed to detect a medium effect size (0.30) is of *N* = 63 participants (linear multiple regression). We had 117 participants, so we believe the study meets these power requirements.

### Materials, Procedure, and Scoring

The following self-questionnaires were collectively administered to the children at school. Participants were asked to indicate how frequently they adopted specific behaviors on a five-point Likert scale (never, rarely, sometimes, often, and always).

### Eight Roles Questionnaire

This instrument comprises 24 items (three *per* role) describing the behaviors typically associated with the different bullying roles. Confirmatory analyses, conducted for both self-report (Belacchi, 2008) and teacher report versions (Belacchi and Farina, 2010) of the Eight Roles Questionnaire, suggested a latent structure composed of four macro-roles: Hostile Roles (Bully, Assistant, and Reinforcer), Pro-social Roles (Defender, Consoler, and Mediator), Victim, and Outsider. Participants are assigned a mean score for each of the eight roles, as well as for both the hostile and pro-social macro-roles.

Reliability Analyses showed the following indices: *α* = 0.900 (Hostile Roles); *α* = 0.833 (Prosocial Roles); *α* = 0.608 Victim; and *α* = 0.535 Outsider, confirming the values evidenced in the previous studies (Belacchi, 2008; Belacchi and Farina, 2010).

### Empathic Responsiveness Scale

The modified version (Belacchi, 2008) of the Interpersonal Reactivity Index (IRI) by Davis (1980) comprises *Perspective Taking* (PT) and *Empathic Concern* (EC) subscales, respectively, assessing the cognitive ability to take the perspective of others and affective reactions to others’ distress. Children received a score from 1 to 5 for: EC (seven item), PT (seven items), and Total Empathy (14 items). The reliability indexes of Empathy Scale measures are, respectively: *α* = 0.603 (EC), *α* = 0.44 (PT), and *α* = 0.671 (Total Empathy).

### Definitional, Metalinguistic Task

The task, individually administered in a quiet room at school, comprises six answer levels. Children were asked to define a list of 32 words: eight nouns, eight adjectives, eight verbs (four concrete and four abstract, respectively; Belacchi and Benelli, 2017) and eight terms describing the main emotions: four primary (*happiness*, *sadness*, *fear*, and *anger*) and four secondary (*shame*, *guilt*, *envy*, and *pride*) ones. The reliability indexes of Definitional Task measures were: *α* = 0.913 (Total Definition), *α* = 0.841 (Primary Emotion Definition), and *α* = 0.759 (Secondary Emotion Definition).

The words were presented in random order, introduced by the question: “Can you tell me what the word … ‘xxx’ … means?.” Each participant received mean definitional scores (range 0–5) for total words, primary, and secondary emotion terms (see Table 1). The criteria for the construction of the scale and attribution of answers to the levels are reported in details in Belacchi and Benelli (2007, 2017).

## Results

Previous studies using the definitional scale (Belacchi and Benelli, 2007, 2017) did reveal differences in definitional competence according to age but not according to gender. Since the present study is based on correlational measures, this last variable was not analyzed in the other two psychological dimensions (empathy and roles) and not included in the research design.

As regards the effects of age on empathy and roles, again age was not included because the literature on these issues is still controversial and because the role of age in these fields was not the focus of the study.

### Relationships Between Empathy and Definitional Competence

According to the first hypothesis, correlations were calculated between the Definitional scores and Empathy measures (see Table 2). Results showed a significant positive association between definitional competence and all the empathy measures, supporting the expectation.

**Table 2:** Partial correlation (controlling for age) between Definitional and Empathy scores.

Definitional Measures	Total Empathy	Empathic Concern	Perspective Taking
Total Definitions	0.400^***^	0.358^***^	0.331^***^
Primary Emotion Definitions	0.279^**^	0.285^**^	0.193^*^
Secondary Emotion Definitions	0.272^**^	0.274^**^	0.194^*^

*** *p* < 0.001

** *p* < 0.01

* *p* < 0.05.

### Age Related Trends in Definitional Competence

As regards the second hypothesis, a univariate ANOVA analysis on definitional scores for age groups (8–10 years old) was performed. Results showed a significant influence of age [*F*(2, 116) = 6,075, *p* = 0.003]. The oldest group 2.80 (*SD* = 0.52) is significantly better (*p* < 0.05) than both the younger groups: 2.41 (0.41), *d* = 0.64; 2.39 (*SD* = 0.75), *d* = 0.83, respectively, which did not differ significantly from each other (*d* = 0.04).

### Identification of Social Roles

In the first place, the interpersonal structure of the participant groups was identified by means of correlations among the scores for the different bullying roles, as a preliminary step to relate definitional competence and empathy with the social roles.

Results confirmed previous literature (Belacchi, 2008; Belacchi and Farina, 2010): Hostile Roles were positively correlated with one another: Bully with Assistant (*r* = 0.717, *p* < 0.001), Bully with Reinforcer (*r* = 0.712, *p* < 0.001), Assistant with Reinforcer (*r* = 0.691, *p* < 0.001); Pro-social Roles were also inter-correlated: Defender with Consoler (*r* = 0.731, *p* < 0.001); Defender with Mediator (*r* = 0.586, *p* < 0.001), Consoler with Mediator (*r* = 0.659, *p* < 0.001); both Victim and Outsider displayed a closer association with hostile roles (respectively, with Bully: *r* = 0.371, *p* < 0.001, *r* = 0.292, *p* < 0.01; with Assistant: *r* = 0.266, *p* < 0.05, *r* = 0.315, *p* < 0.001; with Reinforcer: *r* = 0.305, *p* < 0.001, *r* = 0.330, *p* < 0.001) than with pro-social roles. The Outsider showed a significantly negative association both with Bully (*r* = −0.204, *p* < 0.05) and Assistant (*r* = −0.271, *p* < 0.01), confirming its pro-bullying disposition (Belacchi, 2008; Belacchi and Farina, 2010). Hence, all subsequent correlational analyses were performed on the four macro-roles.

### Relationships Among Definitional Competence, Empathy, and Roles in Bullying

To verify the relationship between empathy measures and definitional ability, in different bullying roles, correlations, controlling for age, were performed (see Table 3). The hostile macro-role was negatively associated with all the definitional measures: mean total score (*r* = −0.471, *p* < 0.001) and emotion term scores for both the primary (*r* = −0.277, *p* < 0.01) and secondary (*r* = −0.288, *p* < 0.01) emotions. No significant correlations were found with the other macro-roles.

**Table 3:** Partial correlations (controlling for age) between Definitional competence, Empathy, and Macro-roles.

	Hostile Roles	Pro-social Roles	Victim	Outsider
Total Definitions	−0.471^***^	0.076	−0.075	−0.133
Primary Emotion Definitions	−0.277^**^	0.069	−0.023	−0.120
Secondary Emotion Definitions	−0.288^**^	−0.083	−0.065	−0.075
Total Empathy	−0.432^***^	0.417^***^	−0.020	−0.193
Empathic Concern	−0.427^***^	0.361^**^	−0.080	0.226^*^
Perspective Taking	−0.321^**^	0.361^**^	−0.048	−0.107

*** *p* < 0.001

** *p* < 0.01

* *p* < 0.05.

Separate analyses conducted for each of the three hostile roles revealed that the negative relationship between these roles and total definitional performance was stronger for the Assistant (*r* = −0.469, *p* < 0.001) and the Reinforcer (*r* = −0.448, *p* < 0.001) than for the Bully (*r* = −0.366, *p* < 0.001). Similarly, for primary emotions, the correlations were as follows: Assistant, *r* = −0.298, *p* < 0.01; Reinforcer, *r* = −0.258, *p* < 0.01; and Bully, *r* = −0.198, *p* < 0.05. For secondary emotions, they were: Assistant, *r* = −0.287, *p* < 0.01; Reinforcer, *r* = −0.306, *p* < 0.01; and Bully, *r* = −0.218, *p* < 0.05.

### Relationships Between Empathy and Social Roles

With regard to the fourth hypothesis, the hostile macro-role was negatively correlated with both two empathy measures (Empathic Concern and Perspective Taking), whereas the pro-social macro-role was positively correlated. The Outsider showed a negative correlation with the measure of Empathic Concern. No correlation was found with the Victim role.

### Regression Analysis

In order to ascertain the relative influence of definitional competence and empathic disposition in hostile behaviors, separate step-wise regression analyses were conducted, with total definitional and total empathy scores as predictors and the hostile macro-role and each of the single hostile roles as dependent variable, in turn (Table 4).

**Table 4:** Stepwise Regression Analysis of Total Definitional Competence and Total Empathy Scores on Hostile Role Measures (macro and single).

	*R*	*R*^2^	Beta	*t*	*p*
**Hostile Roles**					
Total Definitions	0.395	0.156	−0.395	−4.363	0.001
Total Empathy	0.465	0.216	−0.270	−2.804	0.006
**Bully**					
Total Empathy	0.354	0.125	−0.354	−3.893	0.001
**Assistant**					
Total Definitions	0.372	0.138	−0.372	−4.124	0.001
**Reinforcer**					
Total Definitions	0.377	0.142	−0.271	−2.808	0.006
Total Empathy	0.442	0.196	−0.254	−2.630	0.010

For the hostile macro-role, definitional competence was the main predictor (accounting for about 16% of variance), followed by empathy (explaining a further 6% of variance). In the case of the Bully, the sole predictor was empathy (accounting for 12% of variance); in the case of the Assistant, the sole predictor was definitional competence (explaining about 14% of variance); in the case of the Reinforcer, the main predictor was definitional competence (accounting for 14% of variance), the second was empathy (explaining a further 5% of variance).

## Discussion and Conclusions

The main aim of the present study was to analyze the inverse relationships of the disposition to adopt hostile roles with the ability to define words. The underlying reason was to identify a common meta-representational component in both empathic disposition and definitional competence, by showing that a lack of this higher-order trait may lead to perform hostile interpersonal behaviors.

Firstly, the negative correlation between definitional competence and hostile roles confirms the hypothesis that a strong disposition to engage in aggressive conduct is linked to poor metalinguistic ability to define words, especially emotion terms. No correlations between definitional abilities and the other roles were found.

Secondly, the correlations between definitional scores and empathy measures confirmed that both defining and empathizing are underpinned by the ability to apply a meta-level of analysis: to the linguistic and to the interpersonal information, respectively. Even though other authors have particularly stressed the cognitive components of the meta-level capacity to understand others’ states of mind (Carruthers, 2009), in our data, this relationship holds for both the cognitive and affective dimensions of empathy, probably because both these dimensions go beyond elementary forms of emotional contagion and require the cognition of the self (Lewis, 2002).

Concerning the empathic dimension, the positive correlation between pro-social roles and empathy is in accordance with previous evidence that the capacity to understand others’ emotions and needs is required to defend or help peers in distress (Warden and MacKinnon, 2003).

Currently, the contribution of meta-representational abilities to the different roles involved in bullying remains controversial; for example, Monks et al. (2005) did not find superior meta-representational ToM abilities in bullies, whereas Gini (2006) found positive associations between ToM and hostile roles. Sania et al. (2012) found that a poor ToM in early childhood of bullies, victims, and bully-victims increased the risk of becoming victims and bully-victims in early adolescence. Gini (2006) and Caravita et al. (2010) found that good Theory of Mind skills were positively linked to defending behaviors, in primary school children and in young adolescents, respectively.

Our correlational results suggest that poor metalinguistic skills are more strongly associated with the secondary hostile roles (Assistant, Reinforcer) than with the dominant Bully. The aggressive behaviors of Bullies are attributable to poor empathic disposition and not to poor meta-representational ability (as reflected in definitional scores); in contrast, the active contribution of the Assistant to violent behaviors is directly linked to a lack of meta-representational skill (definitional competence as the sole, negative, and predictor); this also holds for the Reinforcer, which however is also characterized by low empathy (definitional competence as the main predictor and empathy as the second, both negative). This is consistent with the literature on the leadership behaviors: according to the *cognitive and social skills model* (Sutton et al., 1999), bullies are not socially incompetent actors, but manipulative individuals seeking personal advantage and recruiting dependent followers. In contrast, the other two aggressive but secondary roles are associated with more limited linguistic and reflective capacities.

Despite these internal differences, hostile roles are characterized by poor definitional abilities; this relationship may be accounted for by the metalinguistic nature of definitions and, more generally, by the role of language in personal and social-relational development. Other studies have already demonstrated the involvement of language in controlling impulses and behavioral reactions. Vallotton and Ayoub (2011), analyzing data on vocabulary, talkativeness, and self-regulation, between 14 and 36 months, found that gains in self-regulation were best predicted by gains in lexicon, even after controlling for cognitive ability. Cuskelly et al. (2003) found that receptive language was positively associated with the capacity to delay gratification, at least in typically developing groups. Nippold and Ward-Lonergan (2010), and Nippold et al. (2008) showed that good verbal reasoning and syntactic abilities (such as those required to produce canonical definitions) are crucial in argumentative and expository tasks, particularly those involving controversies, because such skills are required to mentally devise alternative scenarios or future outcomes and avoid immediate and simplistic behavioral reactions.

In synthesis, the novelty of our contribution is showing that poor meta-representational skills, as reflected in children’s levels of empathy and definitional competence, play a relevant influence on the tendency to adopt hostile roles: aggressive individuals, due to their poorer ability to reflect in more abstract and detached ways and their greater proneness to more immediate and concrete solutions (Crick and Dodge, 1994), seem less sensitive to collaborative principles and conventional rules, in both the linguistic and personal-interpersonal domains.

Some issues require further investigation: replicating this study with larger groups, instruments other than self-report questionnaires (for a recent discussion of the strengths and weakness of self-report measures in bullying, see Jetelina et al., 2019), and considering also the role of other psychological dimensions and measures, such as moral disengagement and emotion comprehension, will provide further information about how cognitive, emotional, moral development, sociability, and metalinguistic skills interact to determine interpersonal behaviors. Indeed, linguistic and emotional factors and pro-sociality appear to act jointly, rather than separately, to regulate social interaction (Bakopoulou and Dockrell, 2015). Moreover, only a longitudinal research design could really identify possible causal relationships of linguistic and metalinguistic skills with social behavior.

The present findings bear implications for educational intervention to reduce aggressive conduct and foster reflective and cooperative behavior. Possible forms of teaching intervention might focus more closely on language: for example, teaching children to analytically break down the word semantic contents and rearrange them into the conventional definitions (Marinellie, 2010) may be an effective means to develop more general meta-representational abilities, which can be transferred to multiple areas of individual and social life. Another educational strategy could be teaching a foreign language beyond formal academic systems in order to prevent bullying (de la Hoz Martines and Alcantud, 2018), by fostering better problem solving abilities and abstract thinking skills. It is well known that bilinguals show higher metalinguistic abilities from very early on, both in the control of linguistic processing and in the analysis of linguistic knowledge (Cromdal, 1999) particularly in the concept of what a word is (Bialystok, 1987).

In conclusion, promoting abilities in definitional skills could foster not only the tendency to assume other-centered perspectives and adhere to conventionally shared criteria, but also particularly to better analyze and express one’s own emotional needs and thoughts, as prerequisite to negotiate and resolve conflicts with peers.

## Data Availability Statement

The raw data supporting the conclusions of this article will be made available by the authors, without undue reservation.

## Ethics Statement

Ethical review and approval was not required for the study on human participants in accordance with the local legislation and institutional requirements. Written informed consent to participate in this study was provided by the participants’ legal guardian/next of kin.

## Author Contributions

CB: Concept of the study, statistical analysis, data collection and interpretation, and writing of the article. BB: Background of the study, data interpretation, and writing of the article. Both the authors contributed to the article and approved the submitted version.

### Conflict of Interest

The authors declare that the research was conducted in the absence of any commercial or financial relationships that could be construed as a potential conflict of interest.
